# Re-Audit of the Assessment and Prevention of Risk of Fragility/Osteoporotic Fractures by Community Physicians in at Risk Old-Age Female Patients Admitted to Central Norfolk Older Adult Inpatient Unit at the Norfolk and Suffolk NHS Foundation Trust

**DOI:** 10.1192/bjo.2025.10666

**Published:** 2025-06-20

**Authors:** Samuel Diduyemi, Amina Jaji, Roseline Abiola Samuel

**Affiliations:** Norfolk and Suffolk NHS Foundation Trust, Norwich, United Kingdom

## Abstract

**Aims:** Osteoporotic fractures are a major cause of mortality and morbidity in older adults, often leading to long-term disability and reduced quality of life. The National Institute for Health and Care Excellence (NICE) guidelines (2012) recommend that women aged ≥65 years and men aged ≥75 years be assessed for fracture risk using tools such as FRAX and QFracture. These assessments help identify high-risk individuals, enabling timely interventions like lifestyle changes, bone density scans (DEXA), and pharmacological treatments, including calcium and bisphosphonates, to prevent fractures. This audit aims to assess compliance with these NICE guidelines by General Practitioners (GPs) in a hospital setting and determine whether high-risk patients received appropriate management.

**Methods:** Retrospective audit was conducted using electronic patient records. The sample included 20 patients admitted to the ward in the month of December 2024. The audit focused on determining whether fracture risk assessments were performed using FRAX or QFracture for service users (women) aged 65 years and older. Additionally, the audit examined whether high-risk patients received appropriate management, including bone density scans (DEXA), lifestyle changes, and pharmacological treatments. Secondary risk factors such as chronic kidney disease, diabetes, and smoking were also evaluated for their impact on fracture risk.

**Results:** 0% had documented FRAX/QFracture assessments or evidence of a DEXA scan.

Over 90% of the audited patients were classified as having intermediate or high risk for major osteoporotic or hip fractures within 10 years.

Despite the high fracture risk, only 19% of patients were actively treated with bone-protecting medications, and none had documented assessments for fragility fracture risk prior to admission.

60% of the patients had secondary risk factors, such as chronic kidney disease, diabetes mellitus, rheumatoid arthritis, glucocorticoid use, smoking, or low BMI, which further increased their fracture risk.

Approximately 70% had a history of falls, and 57% had a history of previous fractures. However, none had undergone orthopaedic surgeries.

Internal barriers to initiating bone-protecting medications, largely due to the mental health-focused nature of the care team, were noted, with responsibility for prescribing remaining with GPs.

**Conclusion:** The audit revealed significant gaps in the implementation of NICE guidelines, with none of the patients receiving documented fracture risk assessments or appropriate interventions. Despite a high prevalence of secondary risk factors and fall histories, the management of fracture risk was insufficient. Addressing internal barriers and improving follow-up care is critical to ensuring better adherence to guidelines and preventing fragility fractures in high-risk patients.

